# 3D Hierarchically Structured Tin Oxide and Iron Oxide-Embedded Carbon Nanofiber with Outermost Polypyrrole Layer for High-Performance Asymmetric Supercapacitor

**DOI:** 10.3390/nano13101614

**Published:** 2023-05-11

**Authors:** Chang-Min Yoon, Suk Jekal, Dong-Hyun Kim, Jungchul Noh, Jiwon Kim, Ha-Yeong Kim, Chan-Gyo Kim, Yeon-Ryong Chu, Won-Chun Oh

**Affiliations:** 1 Department of Chemical and Biological Engineering, Hanbat National University, Daejeon 34158, Republic of Korea; 2 McKetta Department of Chemical Engineering and Texas Material Institute, The University of Texas at Austin, Austin, TX 78712, USA; 3 Department of Advanced Materials Science and Engineering, Hanseo University, Seosan-si 31962, Republic of Korea

**Keywords:** 3D hierarchical structure, supercapacitor, tin oxide, iron oxide, polypyrrole

## Abstract

Herein, unique three-dimensional (3D) hierarchically structured carbon nanofiber (CNF)/metal oxide/conducting polymer composite materials were successfully synthesized by combinations of various experimental methods. Firstly, base CNFs were synthesized by carbonization of electrospun PAN/PVP fibers to attain electric double-layer capacitor (EDLC) characteristics. To further enhance the capacitance, tin oxide (SnO_2_) and iron oxide (Fe_2_O_3_) were coated onto the CNFs via facile hydrothermal treatment. Finally, polypyrrole (PPy) was introduced as the outermost layer by a dispersion polymerization method under static condition to obtain 3D-structured CNF/SnO_2_/PPy and CNF/Fe_2_O_3_/PPy materials. With each synthesis step, the morphology and dimension of materials were transformed, which also added the benign characteristic for supercapacitor application. For the practical application, as-synthesized CNF/SnO_2_/PPy and CNF/Fe_2_O_3_/PPy were applied as active materials for supercapacitor electrodes, and superb specific capacitances of 508.1 and 426.8 F g^−1^ (at 1 A g^−1^) were obtained (three-electrode system). Furthermore, an asymmetric supercapacitor (ASC) device was assembled using CNF/SnO_2_/PPy as the positive electrode and CNF/Fe_2_O_3_/PPy as the negative electrode. The resulting CNF/SnO_2_/PPy//CNF/Fe_2_O_3_/PPy device exhibited excellent specific capacitance of 101.2 F g^−1^ (at 1 A g^−1^). Notably, the ASC device displayed a long-term cyclability (at 2000 cycles) with a retention rate of 81.1%, compared to a CNF/SnO_2_//CNF/Fe_2_O_3_ device of 70.3% without an outermost PPy layer. By introducing the outermost PPy layer, metal oxide detachment from CNFs were prevented to facilitate long-term cyclability of electrodes. Accordingly, this study provides an effective method for manufacturing a high-performance and stable supercapacitor by utilizing unique 3D hierarchical materials, comprised of CNF, metal oxide, and conducting polymer.

## 1. Introduction

A supercapacitor is one type of energy storage device with characteristics in between those of conventional capacitors and batteries [1,2]. Conventional capacitors possess the advantages of high-power density, but suffer from a shortage of energy density [3]. In contrast, batteries possess a low-power density; however, batteries are designed to exhibit a sustainable energy density, which is useful in a variety of fields [4]. Meanwhile, supercapacitors are receiving wide interest, due to their characteristics of high-power density and stability, which can compensate for the insufficient part of conventional capacitors and batteries [5]. For the classification of supercapacitors, there are electric double-layer capacitors (EDLCs) and pseudo-supercapacitors. Also, combinations of EDLCs and pseudo types can attain hybrid types of supercapacitors. Specifically, carbon and carbonaceous materials are typical active materials for EDLCs, which physically store charges by the adsorption and desorption of ions without any chemical reactions [6,7].

On the other hand, pseudo-supercapacitors store electrical energies by redox reactions of active materials, resulting in faradaic charge transfer, which differs from the operating mechanism of EDLCs [8]. Various metal oxides, such as Fe_2_O_3_, MoO_3_, SnO_2_, and V_2_O_5_, can be employed as active materials for pseudo-supercapacitors. While pseudo-capacitors have higher energy densities compared with EDLCs, due to the difference in energy storage mechanisms, they have relatively lower cyclability, stability, and rate capability than EDLCs, owing to their charging/discharging mechanisms involving chemical reactions [9,10]. Moreover, hybrid types of supercapacitors can be prepared by integrating carbonaceous materials with metal oxides to add up the positive natures of EDLCs and pseudo-supercapacitors [11,12,13].

Electrospinning is one of the most convenient methods for preparing fiber-like materials. Specifically, carbon nanofibers (CNFs) can be readily synthesized by electrospinning polymer-dissolved solutions, such as polyacrylonitrile (PAN) and polyvinylpyrrolidone (PVP), followed by carbonization [14,15]. In perspective of capacitive materials, CNFs have various advantages arising from their one-dimensional (1D) structure, including high specific surface area, electrical conductivity, and ion flux between interfaces [16,17,18]. Additionally, pseudo-capacitance can be easily added to CNFs by mixing metal oxide precursors with polymer-dissolved solutions prior to carbonization or introducing thereafter. With the addition of metal oxide, 1D CNFs turn into two-dimensional (2D) materials. In addition, controlled growth of metal oxides, including needle-like, flower-like, or further addition of materials, results in three-dimensional (3D) hierarchically structured materials [19,20].

The 3D hierarchically structured materials possess various advantages over 1D or 2D materials, such as high specific area, accessibility of electrolyte on the active site, and surface-to-volume ratio, which result in an enormous increment in electrochemical performance [21,22,23]. However, there are some drawbacks, such as difficulties in synthesis, fragility of the product, and degradation upon the charging and discharging process [24,25,26,27]. One typical method to overcome such limitations is to introduce the outermost conducting polymer layer on the 3D materials. Some previous studies have successfully reported the prevention effect on 3D-structured active materials during the electrochemical reaction via introducing polypyrrole (PPy), polyaniline (PANI), and poly(3,4-ethylenedioxythiophene) (PEDOT) layers [28,29,30]. However, the polymerization methods described in previous studies utilize the vapor deposition polymerization (VDP) or electrochemical polymerization, which are rather complicated methods with low yields. In this regard, there is a necessity for employing a relatively simple dispersion polymerization method to protect the 3D-structured hierarchical materials for supercapacitor application.

In this study, 3D hierarchically structured carbon nanofiber/metal oxide/conducting polymer materials were synthesized for application in highly stable asymmetric supercapacitor (ASC) devices. Firstly, 1D CNFs were prepared by the electrospinning method using a PAN/PVP solution, and the carbonization process was followed. To achieve high electrochemical performance, SnO_2_ and Fe_2_O_3_ were deposited onto CNFs using a facile hydrothermal treatment to obtain CNF/SnO_2_ and CNF/Fe_2_O_3_ materials with unique structures. Finally, the outermost PPy layer was coated onto the surfaces of the CNF/SnO_2_ and CNF/Fe_2_O_3_ materials via dispersion polymerization under static conditions to obtain 3D hierarchically structured CNF/SnO_2_/PPy and CNF/Fe_2_O_3_/PPy materials. The CNF/SnO_2_/PPy- and CNF/Fe_2_O_3_/PPy-based electrodes exhibited specific capacitances of 508.1 and 426.8 F g^−1^ at a current density of 1 A g^−1^, respectively. The as-fabricated all-solid-state ASC device, using CNF/SnO_2_/PPy-based electrodes on the positive side and CNF/Fe_2_O_3_/PPy-based electrodes on the negative side, manifested a remarkable specific capacitance of 101.2 F g^−1^ at 1 A g^−1^, with an expanded voltage window up to 2.0 V. Moreover, the ASC device manifested an excellent 81.1% retention rate after 2000 cycles of charge/discharge. Such excellent electrochemical performances and stabilities were ascribed to the successful formation of 3D hierarchical structures of EDLC CNF, pseudo-capacitive metal oxides (SnO_2_ and Fe_2_O_3_), and the PPy outermost layer, and their synergistic contributions. Hence, designing a 3D hierarchically structured material with an outermost conducting polymer layer may provide a new possibility for the fabrication of active materials for high-performance hybrid supercapacitors.

## 2. Materials and Methods

### 2.1. Materials

Pyrrole (reagent grade, 98.0%), polyacrylonitrile (PAN, *M_w_* of ca. 150,000), polyvinylpyrrolidone (PVP, *M_w_* of ca. 1,100,000), poly(vinylidene fluoride) (PVDF, *M_w_* of ca. 500,000), poly(vinyl alcohol) (PVA, *M_w_* of ca. 90,000), and 1-Methyl-2-pyrrolidinone (NMP, 99.0%) were obtained from Sigma-Aldrich Co. (Burlington, MA, USA). Sodium sulfate (Na_2_SO_4_, 98.5%) and dimethylformamide (DMF, 99.0%) were acquired from Samchun Chemical Company (Seoul, Republic of Korea). Iron(III) chloride hexahydrate (FeCl_3_·6H_2_O, 99.0%) and tin chloride pentahydrate (SnCl_4_·5H_2_O, 98.0%) were obtained from the Junsei Chemical Co. (Tokyo, Japan). Carbon black was purchased from Thermo Fisher Scientific (Waltham, MA, USA). All chemicals were used as received without any further reaction or treatment.

### 2.2. Preparation of 1D Carbon Nanofibers (CNFs)

In a typical synthesis, the precursor for CNF was prepared by mixing PAN (1.0 g) and PVP (0.8 g) into DMF (13 mL) at 40 °C for 4 h with vigorous stirring. The resulting homogeneous solution was then injected into a syringe (10 mL, tip diameter of 0.5 mm) and placed into the electrospinning device (ESR200, NanoNC, Seoul, Republic of Korea). For the electrospinning conditions, 15 kV voltage was applied with the flow rate of 0.8 mL hr^−1^, maintaining the 15 cm distance between the tip end and collector. The as-spun PAN/PVP nanofibers were dried in the oven at 75 °C for 6 h. The stabilization process was carried out by heating the dried nanofibers in the tube furnace at 250 °C for 2 h (air, heating rate of 2 °C min^−1^). Finally, the carbonization process was followed at 900 °C for 2 h (nitrogen, 5 °C min^−1^) to obtain 1D CNFs.

### 2.3. Fabrication of 2D CNF/SnO_2_ and CNF/Fe_2_O_3_ Materials

To prepare 2D CNF/SnO_2_ and CNF/Fe_2_O_3_ materials, a hydrothermal reaction was conducted to deposit metal oxides of SnO_2_ and Fe_2_O_3_ onto CNFs, respectively. For the preparation of CNF/SnO_2_, precursor solution was prepared by mixing Na_2_SO_4_ (1 mmol) and SnCl_4_·5H_2_O (5 mmol) into DI water (15 mL) with stirring for 2 h for complete dissolution. Then, as-prepared CNFs and homogeneous precursor solution were placed in the stainless-steel autoclave equipped with Teflon reactor for hydrothermal reaction. The hydrothermal reaction was carried for 9 h at 200 °C to acquire CNF/SnO_2_ materials.

Also, the fabrication of CNF/Fe_2_O_3_ was similar to the preparation method of CNF/SnO_2_, but an additional calcination process was conducted. For the preparation of CNF/Fe_2_O_3_, precursor solution was prepared by mixing Na_2_SO_4_ (1 mmol) and FeCl_3_·6H_2_O (1 mmol) into DI water (15 mL) with stirring. Similarly, CNFs and homogeneous precursor solutions were place into autoclave for the hydrothermal reaction at 120 °C for 6 h. After the hydrothermal reaction, resulting CNF/FeOOH materials were collected by the centrifugation, and additional calcination process was conducted in the tube furnace at 400 °C for 1 h (air, heating rate of 2 °C min^−1^) to obtain final CNF/Fe_2_O_3_ materials.

### 2.4. Fabrication of 3D CNF/SnO_2_/PPy and CNF/Fe_2_O_3_/PPy Materials

Three-dimensional CNF/SnO_2_/PPy and CNF/Fe_2_O_3_/PPy materials were prepared by a dispersion polymerization process under static condition. Pyrrole monomer (80 µL) was injected into DI water (10 mL) with stirring. Then, as-synthesized CNF/SnO_2_ and CNF/Fe_2_O_3_ materials were added to pyrrole-dissolved solution and stirred for 40 min. Also, initiator solution was prepared by mixing FeCl_3_·6H_2_O (0.02 mol) into DI water (10 mL). For the PPy polymerization, initiator solution was slowly added to the pyrrole-dissolved solution containing CNF/SnO_2_ and CNF/Fe_2_O_3_ and reaction was processed for 8 h without stirring. The resulting CNF/SnO_2_/PPy and CNF/Fe_2_O_3_/PPy materials were collected by centrifugation and dried in the oven overnight.

### 2.5. Characterization

The morphologies and elemental composition of various CNF-derived materials were investigated by the field emission scanning electron microscope (FE-SEM, S-4800, Hitachi, Tokyo, Japan) instrument, equipped with the energy-dispersive spectrometer add-on (EDS, EX-250, HORIBA Ltd., Kyoto, Japan). Molecular and chemical analysis of CNF-derived materials were conducted by a Fourier-transform infrared (FT-IR) instrument (Nicolet 10, Thermo Fisher, Waltham, MA, USA). The crystallinities of materials were characterized by X-ray diffractometer (XRD, D8 Adv., Bruker Co., Billerica, MA, USA) from 10° to 80° (2 theta). Detailed chemical states of materials were analyzed by X-ray photoelectron spectroscopy (XPS, K-alpha, Thermo Fisher Scientific, Waltham, MA, USA).

### 2.6. Assembly of the All-Solid-State Asymmetric Supercapacitor (ASC) Device

An all-solid-state asymmetric supercapacitor device was assembled by employing CNF/SnO_2_/PPy as the positive electrode and CNF/Fe_2_O_3_/PPy as the negative electrode. Also, solid electrolyte was synthesized by dissolving PVA (2.0 g) in DI water (20 mL) under vigorous stirring at 80 °C until the complete dissolution. Then, 1 M Na_2_SO_4_ was mixed into the PVA solution and stirred for an additional 1 h to attain gel-type of electrolyte. The PVA/Na_2_SO_4_ gel electrolyte was obtained after leaving the solution at room temperature. For the ASC device assembly, the ideal gravimetric ratio of CNF/SnO_2_/PPy and CNF/Fe_2_O_3_/PPy was calculated and set to 0.87 to balance the two electrodes. The gravimetric ratio was determined from the evaluated capacitances in a three-electrode system. PVA/Na_2_SO_4_ gel electrolyte was added to the surfaces of CNF/SnO_2_/PPy- and CNF/Fe_2_O_3_/PPy-based electrodes to allow the formation of a thin layer and stored in a fume hood for 12 h. The fabrication of the ASC device was completed by sandwiching gel-sided CNF/SnO_2_/PPy- and CNF/Fe_2_O_3_/PPy-based electrodes together and fixing the outer surfaces using insulating polyimide film tapes to lessen the contact resistance.

### 2.7. Electrochemical Analysis

The working electrode were prepared by mixing as-synthesized materials, carbon black, and PVDF (mass ratio of 8:1.5:0.5) with slight addition of NMP solution. The resulting paste was then cast on a stainless-steel mesh and dried (80 °C) for 12 h. In addition, a copper wire was attached to the mesh using a drop of silver paste to minimize the resistance between the mesh and wire interfaces. Finally, the working electrode was obtained after drying the electrode for an additional 3 h.

For the electrochemical measurement, as-prepared working electrodes were analyzed by the three-electrode system, employing platinum counter electrode and silver/silver chloride reference electrode (Ag/AgCl). Specifically, three types of electrochemical measurements of cyclic voltammetry (CV) analysis, galvanostatic charge-discharge (GCD) measurement, and electrical impedance (EIS) were conducted by potentiostat. The potentiostat used in this study was the Zive SP1 model (WonATech, Seoul, Republic of Korea). For the CV and GCD measurements, optimized voltage ranges for CNF/SnO_2_/PPy-based electrode was from −0.3 to 0.7 V and CNF/Fe_2_O_3_/PPy-based working electrodes was from −1.0 to 0 V. For the CV measurements, five scan rates from 10 to 200 mV s^−1^, were measured. In the case of GCD measurements, five current densities from 0.5 to 10 A g^−1^ were analyzed. In detail, the applied current densities were computed by dividing the applied current by the mass of the active material on the mesh [31]. Using the formulae described below, the gravimetric capacitances (F g^−1^) were able to be calculated from the CV and GCD curves [32]:

(1) $$C=\frac{1}{M\times \nu \times ∆V}\;\overset{V_{0}+∆V}{\underset{V_{0}}{\int }}IdV\left(\mathrm{from}\;\mathrm{CV}\;\mathrm{curves}\right)$$

(2) $$\;C=\frac{I\times ∆t}{M\times ∆V}\left(\mathrm{from}\;\mathrm{GCD}\;\mathrm{curves}\right)$$

In formulae, given parameters are the gravimetric specific capacitance (*C*), the scan rate (*ν*), the current at discharging process (*I*), the voltage (*V*), the operation voltage window (∆*V*), the discharging time (∆*t*), and the weight of the employed active material (*M*). Furthermore, the CV and GCD curves of the ASC device (i.e., two-electrode system) were similarly measured to the as-mentioned three-electrode system, except widening the voltage range up to 2.0 V. Gravimetric energy density (*E*, Wh kg^−1^) and power densities (*P*, kW kg^−1^) of the ASC device were acquired using the following equations [33]:

(3) $$E=\frac{C\times ∆V^{2}}{2}\times \frac{1000}{3600}$$

(4) $$P=\frac{E}{∆t}\times \frac{3600}{1000}$$

Finally, a 10 mV AC voltage was applied to the device in the frequency range of 10^−2^–10^5^ Hz for the EIS analysis.

## 3. Results and Discussion

### 3.1. Fabrication of CNF/SnO_2_/PPy and CNF/Fe_2_O_3_/PPy

**Figure 1:**
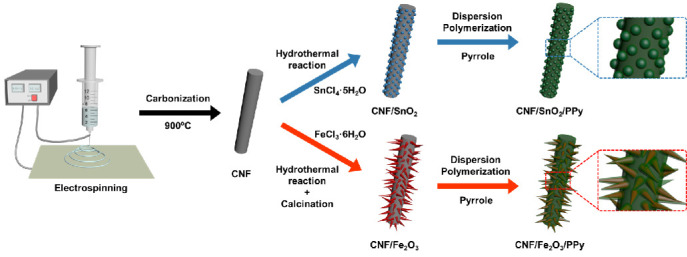
Schematic description for the preparation of 3D hierarchically structured carbon nanofiber (CNF)/SnO_2_/polypyrrole (PPy) and CNF/Fe_2_O_3_/PPy materials.

The overall fabrication process of the 3D hierarchically structured CNF/SnO_2_/PPy and CNF/Fe_2_O_3_/PPy to employ as positive and negative electrodes in supercapacitors is shown in Figure 1. First, 1D CNFs were prepared by electrospinning a PAN/PVP solution, and carbonization followed at 900 °C in nitrogen atmosphere. The deposition of SnO_2_ and Fe_2_O_3_ onto CNFs was then achieved by hydrothermal reaction to form CNF/SnO_2_ and CNF/Fe_2_O_3_ materials, using SnCl_4_·5H_2_O and FeCl_3_·6H_2_O as precursors, respectively. Also, Na_2_SO_4_ was added to control the pH and maintain the shape of the CNFs [34,35]. The metal oxides SnO_2_ and Fe_2_O_3_ were selected for developing high-capacitive materials for positive and negative electrodes, respectively [36]. To prevent the detachment of metal oxides while charging and discharging processes, and to enhance the electrochemical performance, PPy was uniformly introduced to the surface of CNF/SnO_2_ and CNF/Fe_2_O_3_ materials using a dispersion polymerization process under static conditions. This resulted in 3D hierarchically structured materials of CNF/SnO_2_/PPy and CNF/Fe_2_O_3_/PPy.

**Figure 2:**
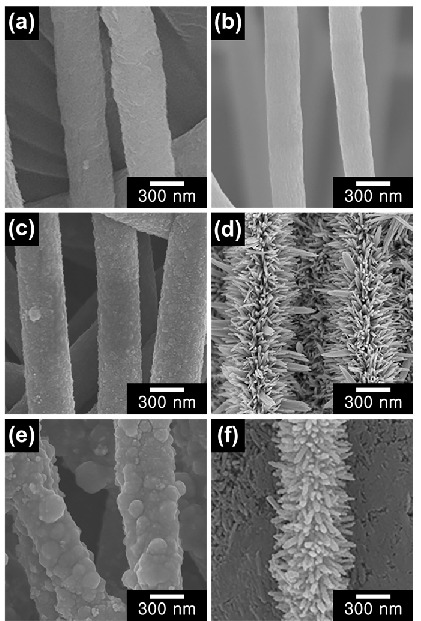
FE-SEM images of (**a**) as-spun PAN/PVP NFs, (**b**) CNFs, (**c**) CNF/SnO_2_, (**d**) CNF/Fe_2_O_3_, (**e**) CNF/SnO_2_/PPy, and (**f**) CNF/Fe_2_O_3_/PPy materials.

The morphologies of the as-spun PAN/PVP NFs, CNFs, CNF/SnO_2_, CNF/Fe_2_O_3_, CNF/SnO_2_/PPy, and CNF/Fe_2_O_3_/PPy were investigated by the FE-SEM analysis (Figure 2). As-spun PAN/PVP NFs were fabricated as uniform 1D cylindrical structures with smooth surfaces and diameters of ca. 410 nm (Figure 2a). After the carbonization process, the CNFs maintained their fibrous structures with a reduced diameter of ca. 300 nm, due to the dehydration reaction of the PAN/PVP NFs (Figure 2b) [37]. The surface of CNF/SnO_2_ was rough, and its diameter was increased to ca. 440 nm, confirming the successful decoration of SnO_2_ to the CNF surface and the formation of 2D-structured materials (Figure 2c) [38]. To investigate the optimal conditions for coating SnO_2_ on CNFs, various CNF/SnO_2_ materials were fabricated via a hydrothermal reaction by varying the amount of SnO_2_ precursor (SnCl_4_·5H_2_O) to 3, 5, and 7 mmol, respectively (Figure S1a–c). With 3 mmol precursor, an inhomogeneous coating of SnO_2_ on the CNFs was achieved. At a precursor concentration of 7 mmol, aggregated states of SnO_2_ were observed, which may degrade the electrochemical performance. Under the experimental conditions, the optimal amount of the precursor was set to 5 mmol for the deposition of SnO_2_, which resulted in a uniform SnO_2_ coating without any uncovered CNFs or aggregation of SnO_2_. In contrast, CNF/Fe_2_O_3_ formed a thorn-like pattern of repeating Fe_2_O_3_ materials on the surface of the CNFs [39]. The length of each thorn-shaped Fe_2_O_3_ material was ca. 250 nm; consequently, the diameter of CNF/Fe_2_O_3_ was ca. 710 nm (Figure 2d). To further examine the growth of Fe_2_O_3_ on the surface of the CNFs, different CNF/Fe_2_O_3_ materials were fabricated by the hydrothermal method by varying amounts of the Fe_2_O_3_ precursor (FeCl_3_·6H_2_O), i.e., 0.5, 1, and 3 mmol (Figure S1d–f). For concentration of 0.5 mmol, Fe_2_O_3_ were unevenly coated on the surface of the CNFs. With 3 mmol precursor, Fe_2_O_3_ particles were enlarged and unable to adhere on the surface of the CNFs. Uniform and controlled growth of Fe_2_O_3_ materials on CNFs were acquired with employing 1 mmol of the precursor.

Also, uniform PPy outermost layer coating on CNF/SnO_2_ and CNF/Fe_2_O_3_ materials were achieved by dispersion polymerization under static condition (Figure 2e,f). In the case of CNF/SnO_2_/PPy, the diameter was increased to ca. 500 nm, verifying the successful coating of outermost PPy of ca. 30 nm (Figure S2a,b). For CNF/Fe_2_O_3_/PPy, the thickness of PPy coating was revealed to be ca. 10 nm, which was determined from the thickness change of the thorn shape (Figure S2c,d). The varying thickness of PPy coating may be ascribed to the volume and shape difference of SnO_2_ and Fe_2_O_3_. Since Fe_2_O_3_ materials grew in a thorn shape with more volume compared with SnO_2_, the thickness of PPy layer was thinner on CNF/Fe_2_O_3_/PPy than CNF/SnO_2_/PPy. Under our experimental condition, detachment of metal oxides was minimized by applying an experimental technique of static polymerization without stirring. To investigate the effect of the polymerization condition, PPy was coated onto 2D materials via a dispersion polymerization under stirring conditions (referred to as CNF/SnO_2_/PPy-stirring and CNF/Fe_2_O_3_/PPy-stirring), and their morphologies were analyzed (Figure S3a,b). As a result, a smaller amount of metal oxides were left on the surface of CNFs for both metal oxides, due to the hydrodynamic generated by the stirring. By dispersing the materials into DI water, it can be observed that the detached metal oxides from the stirring process freely disperse and changing the color of DI water (Figure S4).

The elemental compositions of C, N, O, Sn, and Fe were examined for PAN/PVP NFs, CNFs, CNF/SnO_2_, CNF/Fe_2_O_3_, CNF/SnO_2_/PPy, and CNF/Fe_2_O_3_/PPy, according to the EDS analysis, as listed in Table S1. In the case of PAN/PVP NFs, C, O, and N atoms were detected [40,41]. The proportions of C and O increased for the CNFs, compared with PAN/PVP NFs, whereas the proportion of N decreased, indicating the dehydration reaction of the PAN/PVP NFs [42]. For CNF/SnO_2_ and CNF/Fe_2_O_3_, additional Sn and Fe atoms were revealed, and the proportion of O increased, denoting that SnO_2_ and Fe_2_O_3_ were successfully introduced onto the surfaces of the CNFs. The proportions of C and N atoms increased for CNF/SnO_2_/PPy and CNF/Fe_2_O_3_/PPy, whereas the proportions of Sn, Fe, and O atoms decreased, confirming the successful deposition of the outermost PPy layers on the surfaces of the CNF/SnO_2_ and CNF/Fe_2_O_3_ materials [43].

**Figure 3:**
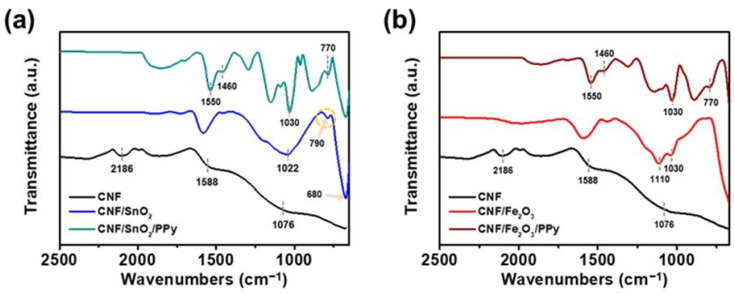
FT-IR spectra of (**a**) CNF/SnO_2_ and CNF/SnO_2_/PPy, and (**b**) CNF/Fe_2_O_3_, and CNF/Fe_2_O_3_/PPy materials. The FT-IR spectrum of CNFs is shown for comparison in (**a**,**b**).

Also, FT-IR was carried out to examine the chemical and molecular structures of CNFs and CNF-derived materials. Figure 3a shows the FT-IR spectra of CNFs, CNF/SnO_2_, and CNF/SnO_2_/PPy. In the case of CNFs, characteristic peaks were detected at 1076 and 2186 cm^−1^, indicating the C–CN and C=NH stretching vibrations [44,45]. Furthermore, the peak around ca. 1590 cm^−1^ was suggestive of the mixed peaks of C=C, C=N, and N–H groups, formed during the CNF carbonization process [46]. For CNF/SnO_2_, characteristic peaks related to SnO_2_ were observed at 680, 790, and 1022 cm^−1^, indicating O–Sn–O bending vibration, Sn–O stretching, and Sn–OH vibration, respectively [47,48]. In the case of CNF/SnO_2_/PPy, additional PPy-related peaks were detected at 770, 1030, 1460, and 1550, matching C–H deformation of the PPy, C–H vibration, C–N stretching, and C=C symmetrical stretching vibration, respectively [49,50,51,52]. SnO_2_-related peaks were maintained in the CNF/SnO_2_/PPy spectra, confirming the successful deposition of PPy without the detachment of SnO_2_ and the development of a 3D hierarchical structure. Figure 3b shows the FT-IR spectra of CNFs, CNF/Fe_2_O_3_, and CNF/Fe_2_O_3_/PPy materials. Characteristic peaks related to Fe_2_O_3_ were detected at 1030 and 1110 cm^−1^, which corresponded to the crystalline Fe–O and Fe–OH vibrations, respectively, confirming the successful incorporation of CNF/Fe_2_O_3_ [53,54]. For CNF/Fe_2_O_3_/PPy, peaks similar to those of PPy were detected, while retaining Fe_2_O_3_-related peaks. Hence, FT-IR analysis confirmed the successful deposition of metal oxides and PPy on the surface of the CNFs.

**Figure 4:**
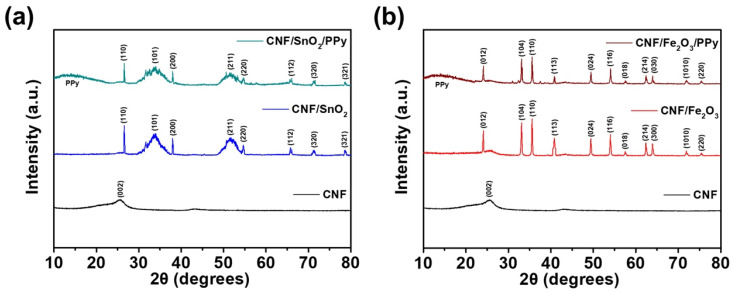
XRD spectra of (**a**) CNF/SnO_2_ and CNF/SnO_2_/PPy, and (**b**) CNF/Fe_2_O_3_, and CNF/Fe_2_O_3_/PPy materials. The XRD spectrum of CNF is shown for comparison in (**a**,**b**).

XRD analysis was conducted to investigate the crystallinities of CNFs and CNF-derived materials (Figure 4). Firstly, a series of CNFs, CNF/SnO_2_, and CNF/SnO_2_/PPy materials were investigated, as shown in Figure 4a. In the case of CNFs, a diffraction peak was detected at 24.2°, which was ascribed to the (002) plane of the amorphous carbon [55]. For CNF/SnO_2_, diffraction peaks were detected at 26.6, 33.9, 38.0, 51.8, 54.7, 65.8, 71.3, and 78.7°, indicating the (110), (101), (200), (211), (220), (112), (320), and (321) crystalline planes of SnO_2_ (JCPDS 41-1445), respectively [56]. For CNF/SnO_2_/PPy, a broad peak between 10° and 20° was observed for the amorphous structure of PPy, whereas the intensities of the CNF/SnO_2_-related peaks diminished, verifying the successful deposition of the outermost PPy layer and the formation of a 3D hierarchical structure [57,58]. Figure 4b presents the XRD spectra of CNFs, CNF/Fe_2_O_3_, and CNF/Fe_2_O_3_/PPy materials. In the case of CNF/Fe_2_O_3_, diffraction peaks were detected at 24.2, 33.1, 35.5, 40.8, 49.4, 54.0, 57.5, 62.3, 63.9, 71.9, and 75.4°, which correspond to the (012), (104), (110), (113), (024), (116), (118), (214), (300), (1010), and (220) planes of the hematite structure of α-Fe_2_O_3_, respectively [59]. Similar to the spectra of CNF/SnO_2_/PPy, the deposition of PPy decreased the peak intensities of CNF/Fe_2_O_3_ and produced an additional broad peak between 10° and 20°. Hence, the XRD patterns of the materials discussed above verified the successful formation of hierarchical structures.

**Figure 5:**
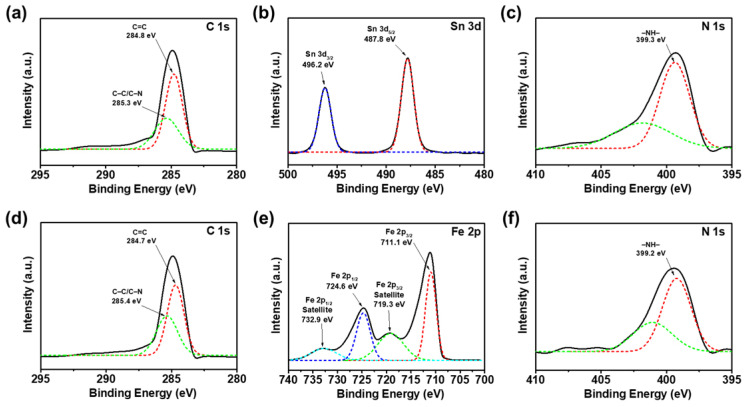
XPS (**a**) C 1s, (**b**) Sn 3d, and (**c**) N 1s spectra of CNF/SnO_2_/PPy materials. XPS (**d**) C 1s, (**e**) Fe 2p, and (**f**) N 1s spectra of CNF/Fe_2_O_3_/PPy materials.

Additional chemical states of CNF/SnO_2_/PPy and CNF/Fe_2_O_3_/PPy materials were evaluated by XPS, as shown in Figure 5. For both materials, it was clearly observed that C 1s spectra were deconvoluted into two peaks at 284.8 and 285.3 eV, which corresponded to the C=C bonds of the CNFs and the C–C/C–N bonds of PPy, respectively [60]. Also, characteristic peaks for Sn and Fe were detected. In the case of Sn, Sn 3d levels were deconvoluted into Sn 3d_5/2_ and Sn 3d_3/2_ at 487.8 and 496.2 eV, indicating the presence of SnO_2_ [61]. For the Fe element, Fe 2p_3/2_ (711.1 eV), Fe 2p_1/2_ (724.6 eV), and satellite peaks were detected at 719.3 and 732.9 eV, which confirmed the successful growth of Fe_2_O_3_ [62,63]. Also, amine groups (–NH–) from the PPy layer was detected from N 1 s spectra at 399.3 eV [64].

### 3.2. Electrochemical Properties of CNF/SnO_2_/PPy- and CNF/Fe_2_O_3_/PPy-Based Electrodes

**Figure 6:**
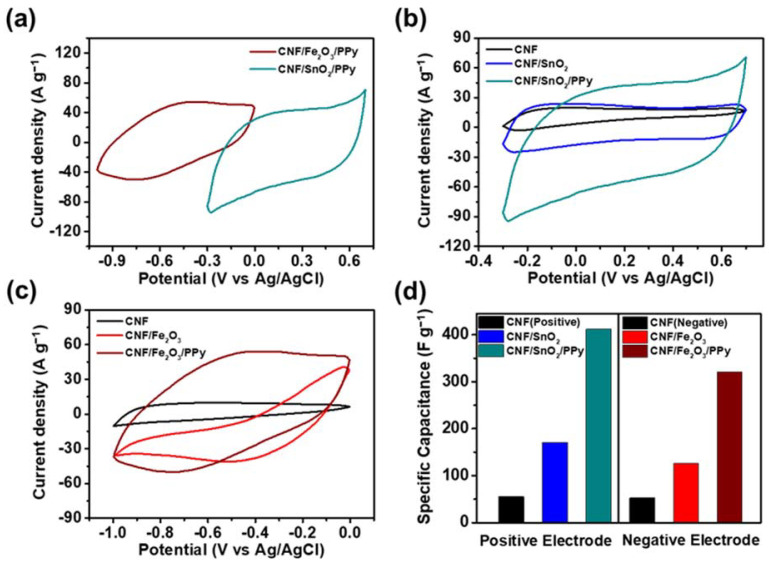
(**a**) The cyclic voltammetry (CV) curves for CNF/SnO_2_/PPy- and CNF/Fe_2_O_3_/PPy-based electrodes at 100 mV s^−1^. (**b**) CV curves of the CNF-, CNF/SnO_2_-, and CNF/SnO_2_/PPy-based electrodes at 100 mV s^−1^ in the potential range from −0.3 to 0.7 V. (**c**) CVs of the CNF-, CNF/Fe_2_O_3_-, and CNF/Fe_2_O_3_/PPy-based electrodes at 100 mV s^−1^ in the potential range of −1.0–0 V. (**d**) Specific capacitances of CNF-, CNF/SnO_2_-, and CNF/SnO_2_/PPy-based electrodes as a positive electrode, and CNF-, CNF/Fe_2_O_3_-, and CNF/Fe_2_O_3_/PPy-based electrodes as a negative electrode.

The electrochemical analysis was conducted under three-electrode measurement, employing CNF/SnO_2_/PPy and CNF/Fe_2_O_3_/PPy as working electrodes for the suitability testing purpose. Figure 6a displays the CV curves of CNF/SnO_2_/PPy- and CNF/Fe_2_O_3_/PPy-based electrodes in the operating potential range of −0.3–0.7 and −1.0–0 V at a 100 mV s^−1^ scan rate, respectively. The different operating voltage range of operating positive and negative electrode materials can expand the operating voltage window in the two-electrode system or assembled supercapacitor device [65,66].

Figure 6b presents the CVs of CNF-, CNF/SnO_2_-, and CNF/SnO_2_/PPy-based electrodes at a 100 mV s^−1^ scan rate over the potential range of −0.3–0.7 V. All CV curves exhibited stable capacitive behavior as supercapacitor electrodes. Noticeably, the area under the CV curve for CNF/SnO_2_/PPy-based electrode was significantly larger than those for CNF- and CNF/SnO_2_-based electrodes, implying higher specific capacitance of the electrode. The evaluated gravimetric specific capacitances of CNF-, CNF/SnO_2_-, and CNF/SnO_2_/PPy-based electrodes were measured to be 55.2, 170.3, and 412.0 F g^−1^. The increase of the specific capacitance for CNF/SnO_2_/PPy-based electrode was ascribed to the successful development of a 3D hierarchical structure. Furthermore, the CV curves of CNF-, CNF/Fe_2_O_3_-, and CNF/Fe_2_O_3_/PPy-based electrodes were examined at a 100 mV s^−1^ scan rate in the potential range from −1.0 to 0 V, as shown in Figure 6c. Similarly, the CNF/Fe_2_O_3_/PPy-based electrode exhibited a larger area, compared with those for CNF- and CNF/SnO_2_-based electrodes, indicating highest specific capacitance. The gravimetric specific capacitances of CNF-, CNF/Fe_2_O_3_-, and CNF/Fe_2_O_3_/PPy-based electrodes evaluated from the CV curves were calculated as 52.5, 126.8, and 335.6 F g^−1^. Figure 6d shows the gravimetric specific capacitances of CNF-, CNF/SnO_2_-, and CNF/SnO_2_/PPy-based electrodes for the positive electrode, and CNF-, CNF/Fe_2_O_3_-, and CNC/Fe_2_O_3_/PPy-based electrodes for the negative electrode. Accordingly, CNF/SnO_2_/PPy- and CNC/Fe_2_O_3_/PPy-based electrodes exhibited enhanced electrochemical performance, owing to the formation of 3D hierarchically structured materials.

**Figure 7:**
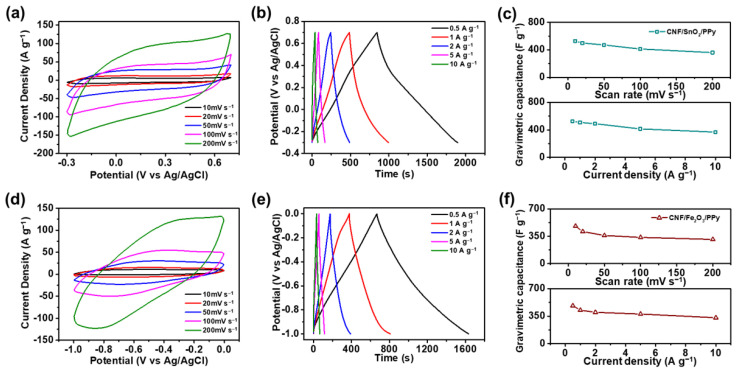
(**a**) CVs and (**b**) Galvanostatic charge-discharge (GCD) curves of CNF/SnO_2_/PPy-based electrode at different scan rates and current densities (voltage range of −0.3–0.7 V). (**c**) Gravimetric capacitances of CNF/SnO_2_/PPy-based electrode computed from CVs (top) and GCDs (bottom). (**d**) CVs and (**e**) GCDs of CNF/Fe_2_O_3_/PPy-based electrode at different scan rates and current densities. (**f**) Gravimetric capacitances of CNF/Fe_2_O_3_/PPy evaluated from CVs (top) and GCDs (bottom).

Figure 7a represents the CV curves of CNF/SnO_2_/PPy-based electrode at scan rates ranging from 10 to 200 mV s^−1^ in the operating potential range of −0.3–0.7 V. The CVs of CNF/SnO_2_/PPy-based electrode maintained a rectangular-shaped curve, even at high scan rates, exhibiting excellent EDLC behavior [67]. The gravimetric specific capacitances of the CNF/SnO_2_/PPy-based electrode at scan rates of 10, 20, 50, 100, and 200 mV s^−1^ were measured as 527.8, 499.3, 470.9, 412.0, and 360.0 F g^−1^, respectively. It was noticeable that 68.2% of the specific capacitance was maintained as the scan rate varied from 10 to 200 mV s^−1^, showing the excellent rate capabilities of the CNF/SnO_2_/PPy-based electrode [68]. To confirm the advantages of the 3D hierarchically structured material of CNF/SnO_2_/PPy as supercapacitor electrodes, CV curves of CNF- and CNF/SnO_2_-based electrodes were further examined at various scan rates (Figure S5a,b). For the CNF-based electrode, the measured specific capacitance was 126.0 F g^−1^ at 10 mV s^−1^; however, 42.2 F g^−1^ was evaluated at 200 mV s^−1^, and only 33.5% of the capacitance was retained. Furthermore, the specific capacitances of CNF/SnO_2_-based electrode at 10 and 200 mV s^−1^ were measured as 349.8 and 140.0 F g^−1^, respectively, and 40.0% of the capacitance was preserved. The rate capabilities of CNF- and CNF/SnO_2_-based electrodes were significantly lower than those of the CNF/SnO_2_/PPy-based electrode, because the formation of hydrogen bonds through the deposition of PPy accelerated the electron transport rate at the SnO_2_/PPy interface, resulting in energy dissipation [69,70]. Therefore, the outstanding capacitive performance of the CNF/SnO_2_/PPy-based electrode was attributed to its rapid redox reactions and charge transport abilities. Moreover, CV curves of CNF/SnO_2_/PPy-stirring-based electrode were further examined to compare the capacitive difference between the static and stirring conditions during the oxidative polymerization process (Figure S5c). At 10 mV s^−1^, the specific capacitance of CNF/SnO_2_/PPy-stirring-based electrode was evaluated as 210.0 F g^−1^, which was 39.8% capacitance compared to CNF/SnO_2_/PPy-based electrode prepared at static condition. Such low capacitance was observed because the external stirring force caused the metal oxides to fall off from the surface of the CNF, and PPy was not uniformly coated, resulting in the increase in electrical resistance at electrolyte–electrode interface, which caused the interruption of charge transportation [71]. Hence, a uniform PPy coating was successfully achieved under static conditions, verifying the improvement in electrochemical performance.

Furthermore, the GCD curves of CNF/SnO_2_/PPy-based electrode were evaluated at various current densities, ranging from 0.5 to 10 A g^−1^, in the potential range from −0.3 to 0.7 V, as shown in Figure 7b. All GCDs indicated outstanding capacitive behaviors. The CNF/SnO_2_/PPy-based electrode manifested gravimetric specific capacitances of 524.9, 508.1, 489.4, 414.2, and 367.4 F g^−1^ at current densities of 0.5, 1, 2, 5, and 10 A g^−1^. Noticeably, 70.0% of the capacitance was preserved at 10 A g^−1^, compared with that of 0.5 A g^−1^, exhibiting outstanding rate capability, owing to the assistance of charge transportation by metal oxides and conducting polymers [72]. In addition, the coulombic efficiency, a significant parameter for evaluating the efficiency of stored energy, was estimated by calculating the ratio of the charging and discharging times [73]. At 1 A g^−1^, the charging and discharging times were measured as 510 and 508 s, respectively, which exhibited an outstanding coulombic efficiency of 99.6%, verifying the remarkable capacitive performance of the supercapacitor electrode [74]. The specific capacitances of the CNF/SnO_2_/PPy-based electrode evaluated from the CVs and GCDs are shown in Figure 7c. Accordingly, the CNF/SnO_2_/PPy-based electrode exhibited excellent electrochemical performance and assisted ion transport, confirming its high capability as a supercapacitor electrode.

To verify the suitability of the CNF/Fe_2_O_3_/PPy-based electrode as a negative electrode, the CV curves were measured at various scan rates, ranging from 10 to 200 mV s^−1^, in the potential range of −1.0–0 V, as shown in Figure 7d. At 10, 20, 50, 100, and 200 mV s^−1^ scan rates, the gravimetric specific capacitances of the CNF/Fe_2_O_3_/PPy-based electrode were measured as 478.7, 409.9, 359.7, 335.6, and 310.2 F g^−1^, respectively. As the scan rate increased, the specific capacitance decreased, because the rate for charge transfer in the electrode surpassed the rate for ion diffusion at the electrode–electrolyte interface [75]. Nonetheless, 64.8% of the specific capacitance was retained at 200 mV s^−1^, compared to that at 10 mV s^−1^, indicating a remarkable rate capability. To verify the structural advantages of the 3D CNF/Fe_2_O_3_/PPy, the CV curves of CNF- and CNF/Fe_2_O_3_-based electrodes in the potential range of −1.0–0 V were examined at different scan rates (Figure S6a,b). The measured specific capacitances of CNF-based electrode were 178.1 and 44.6 F g^−1^ at 10 and 200 mV s^−1^, respectively, and only 25.0% of the capacitance was preserved. The specific capacitances of CNF/Fe_2_O_3_-based electrode at 10 and 200 mV s^−1^ were 305.4 and 66.1 F g^−1^, respectively, in which the rate capability was calculated to be 21.6%. The CNF/Fe_2_O_3_/PPy-based electrode showed higher rate capabilities than CNF- and CNF/Fe_2_O_3_-based electrodes, because the deposition of PPy facilitated charge transport, resulting in enhanced electrochemical performance [76]. To further analyze the differences between the CNF/Fe_2_O_3_/PPy materials fabricated under static and stirring conditions during the dispersion polymerization process, the CV curves of CNF/Fe_2_O_3_/PPy-stirring-based electrode were investigated (Figure S6c). The specific capacitance of CNF/Fe_2_O_3_/PPy-stirring-based electrode evaluated from the CV curves was 270.0 F g^−1^ at 10 mV s^−1^, which was 56.4% capacitance, compared to that of the CNF/Fe_2_O_3_/PPy-based electrode. The Fe_2_O_3_ materials on the surface of the CNF were separated by stirring, and these materials were entangled with PPy, causing resistive behavior, and thereby degrading the electrochemical performance [77]. Therefore, the electrode based on CNF/Fe_2_O_3_/PPy, fabricated under static conditions, exhibited excellent capacitive performance.

Figure 7e presents the GCD curves of the CNF/Fe_2_O_3_/PPy-based electrode at current densities of 0.5 to 10 A g^−1^, with voltage range of −1.0 to 0 V. Non-linear GCD curves were observed, owing to the limited faradaic reaction on the surface of the active material, revealing pseudo-capacitive behavior [78,79]. At 0.5, 1, 2, 5, and 10 A g^−1^, the specific capacitances of CNF/Fe_2_O_3_/PPy-based electrode evaluated from the GCD curves were 482.6, 426.8, 399.1, 377.5, and 330.4 F g^−1^, respectively, and 68.5% of the capacitances were retained, even at 10 A g^−1^, compared with that of 0.5 A g^−1^. The presence of Fe_2_O_3_ facilitated the charge transport of CNF/Fe_2_O_3_/PPy-based electrode as a negative part, which effectively improved the electrochemical efficiency [80]. The charging and discharging times of the CNF/Fe_2_O_3_/PPy-based electrode at 1 A g^−1^ were measured as 433.4 and 426.8 s, in which 98.5% of the coulombic efficiency was evaluated, confirming its exceptional performance as a negative electrode. Figure 7f presents the specific capacitances of the CNF/Fe_2_O_3_/PPy-based electrode, calculated from the CVs and GCDs as functions of scan rate and current density. The results confirmed that the CNF/Fe_2_O_3_/PPy-based electrode exhibited excellent electrochemical performance and high-rate capabilities. In addition, the capacitive performances of the CNF/SnO_2_/PPy- and CNF/Fe_2_O_3_/PPy-based electrodes were compared with previously reported CNF-derived supercapacitor electrode materials including 3D structure in a three-electrode system, as shown in Table S2 [81,82,83,84,85]. Compared with previously reported materials, the CNF/SnO_2_/PPy- and CNF/Fe_2_O_3_/PPy-based electrodes showed better specific capacitances, signifying the successful preparation of 3D hierarchically structured materials.

### 3.3. Electrochemical Performance of the Assembled ASC Device

To examine the practical electrochemical performance, an all-solid-state ASC device was constructed with CNF/SnO_2_/PPy and CNF/Fe_2_O_3_/PPy electrodes. The ASC device was prepared employing CNF/SnO_2_/PPy as the positive electrode, CNF/Fe_2_O_3_/PPy as the negative electrode, and 1 M Na_2_SO_4_-mixed PVA as a solid-type electrolyte. The amount of active materials were determined to equalize the charges on the positive electrode ($Q_{+}$) and negative electrode ($Q_{-}$), which minimized the loss of capacitance [86]. The charges of electrodes can be computed from the following [87]:

(5) Q=C×∆V×M

where, Q indicates the electrode charge, C is the evaluated gravimetric capacitance, ∆V describes the discharging voltage, and M is the mass loaded to each electrode. The gravimetric ratio ($M_{+}/M_{-}$) was calculated by equalizing the positive and negative charge balance ($Q_{+}=Q_{-}$), in which the relationship is shown according to the following relation [88]:

(6) $$\frac{M_{+}}{M_{-}}=\frac{C_{-}\times ∆V_{-}}{C_{+}\times ∆V_{+}}$$

The gravimetric ratios were evaluated from the CV and GCD results of CNF/SnO_2_/PPy- and CNF/Fe_2_O_3_/PPy-based electrodes in the three-electrode system. From the CV results, the gravimetric ratios were calculated as 0.92, 0.85, 0.85, 0.92, and 0.92 at 10, 20, 50, 100, and 200 mV s^−1^ scan rates, respectively. The ratios computed from GCDs were 0.91, 0.83, 0.77, 0.82, and 0.93 at current densities ranging from 0.5 to 10 A g^−1^. From the evaluated gravimetric ratios, the optimal ratio of the ASC device was set to be 0.87, which is the mean of 10 ratios, calculated from the CV and GCD results. Thus, the ASC device was successfully fabricated by assembling the CNF/SnO_2_/PPy- and CNF/Fe_2_O_3_/PPy-based electrodes with the calculated charge balance ratio.

**Figure 8:**
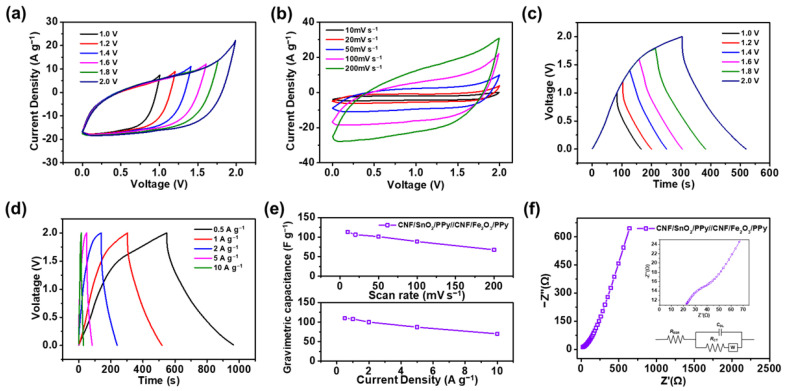
(**a**) CV curves of the CNF/SnO_2_/PPy//CNF/Fe_2_O_3_/PPy ASC device with different voltage windows and (**b**) scan rates. (**c**) GCD curves of device with various voltage windows and (**d**) current densities. (**e**) Gravimetric capacitances of the device calculated from the CVs (top) and GCDs (bottom). (**f**) Electrochemical impedance spectroscopy (EIS) analysis of the ASC device.

The electrochemical analysis of CNF/SnO_2_/PPy//CNF/Fe_2_O_3_/PPy ASC devices was investigated. The CVs of the ASC device were examined at a scan rate of 100 mV s^−1^ in different potential windows of 1.0 to 2.0 V with increments of 0.2 V, as shown in Figure 8a. The operating voltage of the ASC device reached 2.0 V, due to the difference of work functions of metal oxide materials [89]. Unlike alkali and acidic salts, which decrease the operating voltage range by accelerating the decomposition of water, neutral salts, such as Na_2_SO_4_, can effectively secure the voltage range [90]. The amount of the work function difference between electrodes and the electrode surface polarization can conclude the operating voltage window. To prevent the performance degradation over-exceeding 2.0 V, the operating voltage window of the ASC was set to 2.0 V [91,92]. Additionally, the ASC device employing PVA/Na_2_SO_4_ as the gel electrolyte formed a stable quasi-rectangle, resulting in excellent capacitive behavior [93]. Figure 8b presents the CV curves of the device at different scan rates in a voltage range of 0–2.0 V. At all scan rates, quasi-rectangular shapes were formed, while maintaining capacitive behavior. The gravimetric specific capacitances of the ASC device were evaluated as 113.4, 106.4, 101.5, 88.8, and 69.8 F g^−1^ at 10, 20, 50, 100, 200 mV s^−1^, respectively, showing excellent electrochemical performance with a 61.5% rate capability.

The GCD curves of the CNF/SnO_2_/PPy//CNF/Fe_2_O_3_/PPy ASC device were further investigated at 1 A g^−1^ current density in the potential windows of 1.0 to 2.0 V to confirm the capacitive behavior, as shown in Figure 8c. As the voltage range exceeded 2.0 V, the discharging time shortened, owing to polarization, resulting in reduced coulombic efficiency and loss of the main function as an energy storage device [94,95]. Therefore, the optimal voltage range of 2.0 V was determined for GCD investigations. In addition, pseudo-capacitive behavior through rapid redox reactions of the metal oxides was observed in the GCDs, which effectively increased the operating voltage window [96,97]. The measured specific capacitances of the ASC device from the GCDs were 79.4, 80.6, 89.5, 91.3, 94.2, and 101.2 F g^−1^, with coulombic efficiencies of 99.5, 99.1, 98.2, 97.7, 97.4, and 97.2% at 1 A g^−1^ in voltages of 1.0, 1.2, 1.4, 1.6, 1.8, and 2.0 V, respectively. Notably, 97.2% of coulombic efficiency was maintained at 2.0 V, indicating the efficient charge transfer and reversible characteristics of the ASC device. Figure 8d presents the GCDs of the ASC device in the operating voltage window of 2.0 V at different current densities. At 0.5, 1, 2, 5, and 10 A g^−1^ current densities, the gravimetric specific capacitances from the GCD curves were measured as 104.2, 101.2, 99.7, 86.8, and 68.5 F g^−1^, respectively. Approximately 65.7% of the capacitance was effectively maintained at current densities of 0.5 to 10 A g^−1^ ranges. Figure 8e shows the gravimetric specific capacitances of the ASC device evaluated from the CVs and GCDs.

The ion transportation abilities of the CNF/SnO_2_/PPy//CNF/Fe_2_O_3_/PPy ASC devices were evaluated using EIS analysis, as shown in Figure 8f. In addition, an equivalent circuit diagram describing the ASC device was inserted as an inset, which included the resistance of the equivalent series ($R_{\mathrm{ESR}}$), resistance of the charge-transfer ($R_{\mathrm{CT}}$), double-layer capacitance ($C_{\mathrm{DL}}$), and Warburg impedance ($Z_{W}$) [98]. Specifically, $R_{\mathrm{ESR}}$, which is the sum of surface resistances of electrodes and ionic resistance of the electrolyte, was measured as 22.6 Ω in the high frequency region. The $R_{\mathrm{CT}}$ value was determined as 32.2 Ω, which corresponds to the ionic reaction resistance between electrodes and electrolytes. The Warburg impedance was also observed as a straight line in the low-frequency range, depicting excellent capacitive behavior [99]. The as-fabricated CNF/SnO_2_/PPy//CNF/Fe_2_O_3_/PPy ASC device exhibited excellent ion transport capabilities and electrochemical performance, owing to the formation of 3D hierarchical structures.

**Figure 9:**
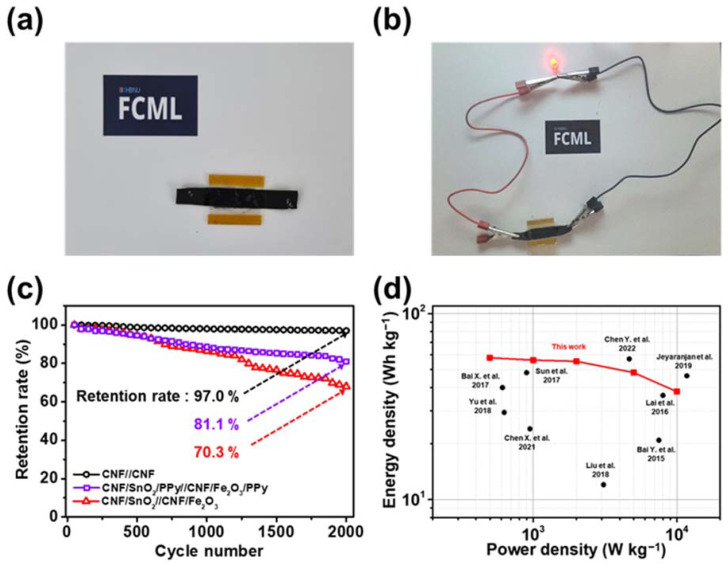
Digital photographs of (**a**) the CNF/SnO_2_/PPy//CNF/Fe_2_O_3_/PPy ASC device and (**b**) the demonstration of the ASC device illuminating the red LED (1.8 V). (**c**) Long-term cyclability test of the ASC device at 5 A g^−1^ for 2000 cycles. (**d**) Ragone plot of the ASC device, compared to other all-solid-state ASC devices. Reproduced by: Sun et al., 2017 [103], Bai X. et al., 2017 [104], Yu et al., 2018 [105], Chen X. et al., 2021 [106], Liu et al., 2018 [107], Bai Y. et al., 2015 [108], Lai et al., 2016 [109], Jeyaranjan et al., 2019 [110], and Chen Y. et al., 2022 [111].

Figure 9a presents a visual image of the CNF/SnO_2_/PPy//CNF/Fe_2_O_3_/PPy ASC device. A PVA/Na_2_SO_4_ gel electrolyte was placed between the electrodes in a sandwich-like configuration. To demonstrate the practical electrochemical application, the ASC device was charged up to 2.0 V and connected to a LED bulb (1.8 V, red) (Figure 9b). The LED bulb was successfully illuminated, denoting the widened voltage window from the incorporation of SnO_2_, Fe_2_O_3_, and PPy. In addition, a cyclability test was conducted for 2000 cycles at 5 A g^−1^ to confirm the stability of the CNF/SnO_2_/PPy//CNF/Fe_2_O_3_/PPy ASC device (Figure 9c). For comparison, the cyclabilities of the ASC (CNF/SnO_2_//CNF/Fe_2_O_3_) and CNF-based EDLC (CNF//CNF) devices were further examined. The CNF//CNF device exhibited the highest retention rate of 97.0% of its initial value, but low specific capacitance, owing to the characteristics of the EDLC [100,101]. The retention rate of the CNF/SnO_2_/PPy//CNF/Fe_2_O_3_//PPy ASC device (81.1%) was higher than that of the CNF/SnO_2_/CNF/Fe_2_O_3_ device (70.3%), owing to the morphological advantages of 3D hierarchically structured materials with the protection of metal oxides. This 3D hierarchically structured morphology maintained long-term cyclability and remarkable specific capacitance of the ASC device, demonstrating the successful composition of metal oxides (SnO_2_ and Fe_2_O_3_) and PPy [102]. Finally, the energy and power densities of the device were calculated and compared with densities of other hierarchically structured all-solid-state ASC devices (Figure 9d) [103,104,105,106,107,108,109,110,111]. The CNF/SnO_2_/PPy//CNF/Fe_2_O_3_//PPy ASC device exhibited energy and power densities of 56.2 Wh kg^−1^ and 0.99 kW kg^−1^ at 1 A g^−1^, respectively. Conclusively, the as-prepared ASC device showed a maximum energy density of 57.9 Wh kg^−1^ (at 0.5 A g^−1^) and a maximum power density of 10.0 kW kg^−1^ at (10 A g^−1^). Compared with other hierarchically structured ASC devices, the device fabricated in this study exhibited outstanding energy and power densities, verifying its excellent performance. Facile deposition of PPy under static conditions was accomplished to prevent the metal oxides from detaching, during which a high-capacitive ASC device was successfully manufactured. Hence, the as-fabricated CNF/SnO_2_/PPy//CNF/Fe_2_O_3_/PPy ASC device with excellent electrochemical performance and cyclability expects to extend the possibility of a new energy storage device for the next generation.

## 4. Conclusions

In summary, 3D hierarchically structured CNF/SnO_2_/PPy and CNF/Fe_2_O_3_/PPy were fabricated as active materials for asymmetric supercapacitor electrodes, using a combination of electrospinning, hydrothermal methods, and dispersion polymerization. The resulting CNF/SnO_2_/PPy- and CNF/Fe_2_O_3_/PPy-based electrodes exhibited outstanding specific capacitances of 508.1 and 426.8 F g^−1^ at a current density of 1 A g^−1^. Such high performance was ascribed to the well-constructed 3D hierarchical structure of CNFs, controlled growth of metal oxides (SnO_2_ and Fe_2_O_3_) decoration, and PPy outermost layer. Each of the synthesis steps have successfully incorporated the EDLCs, pseudo-capacitance, and prevention effect. A practical ASC device assembled by the resulting 3D hierarchically structured materials exhibited superb specific capacitance of 101.2 F g^−1^ (at 1 A g^−1^), with an extended operating voltage window up to 2.0 V. Noticeably, the ASC device displayed an outstanding retention rate of 81.1%, even after 2000 cycles, confirming its long-term stability. This outstanding cyclability was attributed to the deposition of PPy onto the metal oxide-coated CNFs, which prevented the detachment of metal oxides during the charging and discharging processes. The synthetic method suggested in this study may provide a possibility of developing high-performance, yet stable, active materials by utilizing unique 3D hierarchical structures for next-generation supercapacitor applications.

## Data Availability

Data are contained within the article.

## Supplementary Materials

The following supporting information can be downloaded at: https://www.mdpi.com/article/10.3390/nano13101614/s1, Figure S1: FE-SEM micrographs of CNF/SnO_2_ materials using (a) 3 mmol, (b) 5 mmol, and (c) 7 mmol of SnO_2_ precursor (SnCl_4_·5H_2_O), and CNF/Fe_2_O_3_ materials using (d) 0.5 mmol, (e) 1 mmol, and (f) 3 mmol of Fe_2_O_3_ precursor (FeCl_3_·6H_2_O). Figure S2: High-magnitude FE-SEM images of (a) CNF/SnO_2_, (b) CNF/SnO_2_/PPy, (c) CNF/Fe_2_O_3_, and (d) CNF/Fe_2_O_3_/PPy materials. Figure S3: FE-SEM micrographs of (a) CNF/SnO_2_/PPy and (b) CNF/Fe_2_O_3_/PPy materials prepared by the dispersion polymerization with magnetic stirring (referred to as CNF/SnO_2_/PPy-stirring and CNF/Fe_2_O_3_/PPy-stirring). Figure S4: Digital images of (a) CNF/SnO_2_/PPy (left), CNF/Fe_2_O_3_/PPy (right), and (b) CNF/SnO_2_/PPy-stirring (left), CNF/Fe_2_O_3_/PPy-stirring (right) in DI water. Figure S5: CV curves of (a) CNF-, (b) CNF/SnO_2_-, and (c) CNF/SnO_2_/PPy-stirring-based electrodes at various scan rates from 10 to 200 mV s^−1^ in the potential range of −0.3–0.7 V. Figure S6: CV curves of (a) CNF-, (b) CNF/Fe_2_O_3_-, and (c) CNF/Fe_2_O_3_/PPy-stirring-based electrodes at various scan rates from 10 to 200 mV s^−1^ in the potential range of −1.0–0 V.; Table S1: Elemental compositions of PAN/PVP NFs, CNFs, and CNF-derived materials fabricated in this study. Table S2: Comparison of the specific capacitances of CNF/SnO_2_/PPy- and CNF/Fe_2_O_3_/PPy-based electrodes with previous studies.
