# Supplementary material for: 3D Hierarchically Structured Tin Oxide and Iron Oxide-Embedded Carbon Nanofiber with Outermost Polypyrrole Layer for High-Performance Asymmetric Supercapacitor
Source: Nanomaterials (Basel). 2023 May 11;13(10):1614. doi: 10.3390/nano13101614 (PMC10224367; doi:10.3390/nano13101614)
Supplement: Supplementary file 1 [file nanomaterials-13-01614-s001.zip › nanomaterials-2372381-supplementary.pdf]

nanomaterials

Supplementary Materials

3D Hierarchically Structured Tin Oxide and Iron Oxide-Embedded Carbon Nanofiber with Outermost Polypyrrole Layer for High-Performance Asymmetric Supercapacitor

Chang-Min Yoon [1,†], Suk Jekal [1,†], Dong-Hyun Kim [1], Jungchul Noh [2], Jiwon Kim [1], Ha-Yeong Kim [1], Chan-Gyo Kim [1], Yeon-Ryong Chu [1] and Won-Chun Oh [3,*]

[1] Department of Chemical and Biological Engineering, Hanbat National University, Daejeon 34158, Republic of Korea
[2] McKetta Department of Chemical Engineering and Texas Material Institute, The University of Texas at Austin, Austin, TX 78712, USA
[3] Department of Advanced Materials Science and Engineering, Hanseo University, Seosan-si 31962, Republic of Korea
* Correspondence: wc_oh@hanseo.ac.kr; Tel.: +82-41-660-1337; Fax: +82-41-660-1149
† These authors contributed equally to this work.

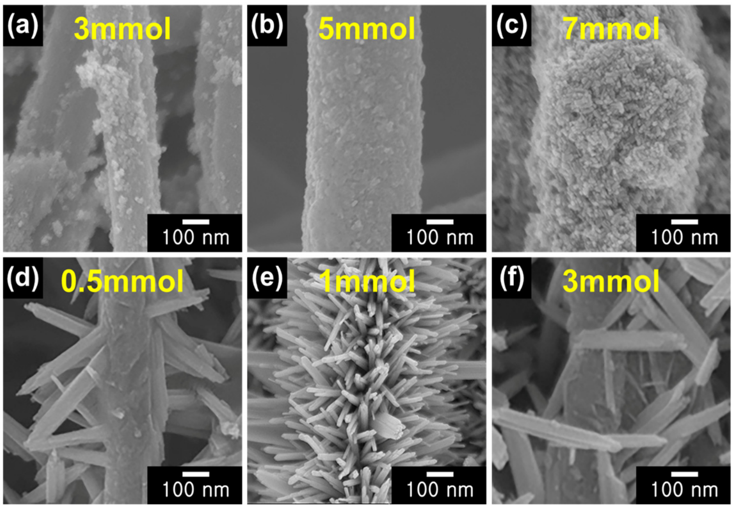

Figure S1. FE-SEM micrographs of CNF/SnO_2 materials using (a) 3 mmol, (b) 5 mmol, and (c) 7 mmol of SnO_2 precursor ($SnCl_4{\cdot}6H_2O$), and CNF/Fe_2O_3 materials using (d) 0.5 mmol, (e) 1 mmol, and (f) 3 mmol of Fe_2O_3 precursor ($FeCl_3{\cdot}6H_2O$).

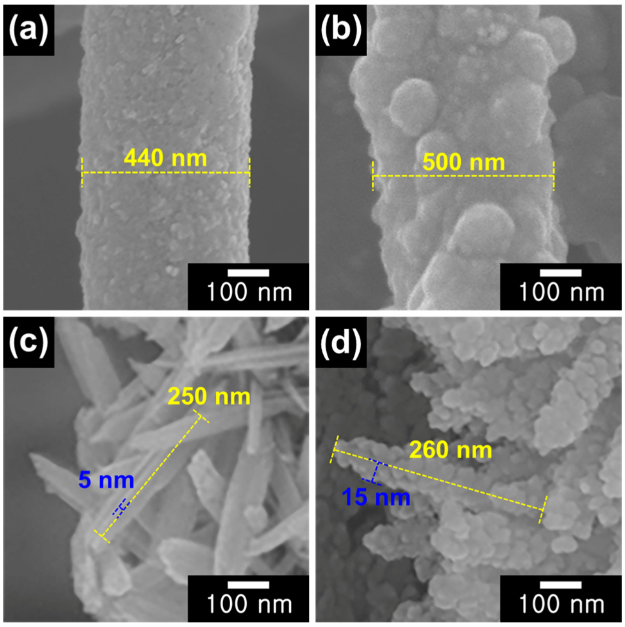

Figure S2. High-magnitude FE-SEM images of (a) CNF/SnO_2, (b) CNF/SnO_2/PPy, (c) CNF/Fe_2O_3, and (d) CNF/Fe_2O_3/PPy materials.

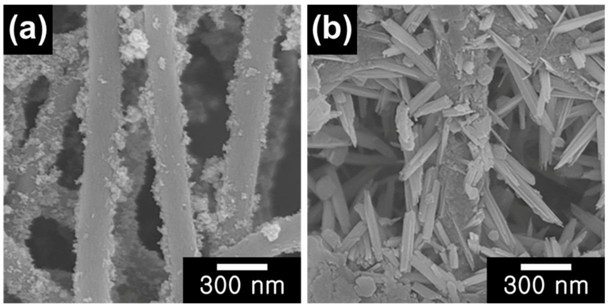

Figure S3. FE-SEM micrographs of (a) CNF/SnO_2/PPy and (b) CNF/Fe_2O_3/PPy materials prepared by the dispersion polymerization with magnetic stirring (referred to as CNF/SnO_2/PPy-stirring and CNF/Fe_2O_3/PPy-stirring).

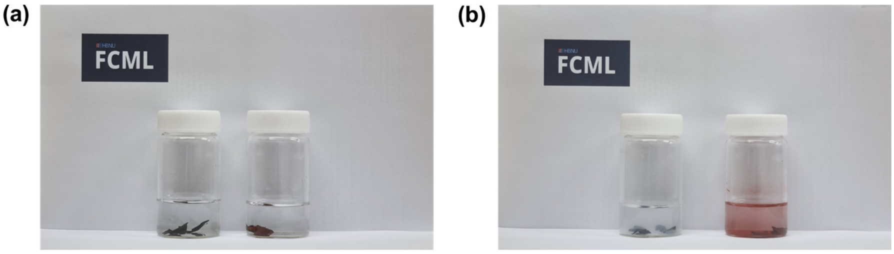

Figure S4. Digital images of (a) CNF/SnO_2/PPy (left), CNF/Fe_2O_3/PPy (right), and (b) CNF/SnO_2/PPy-stirring (left), CNF/Fe_2O_3/PPy-stirring (right) in DI water.

Table S1. Elemental compositions of PAN/PVP NFs, CNFs, and CNF-derived materials fabricated in this study [a].

**Samples**	Element (Atomic %)				
	C	O	N	Sn	Fe
PAN/PVP NFs	84.5	4.3	11.2		
CNFs	87.8	9.0	3.2		
CNF/SnO_2	23.0	18.7	1.5	56.8	
CNF/Fe_2O_3	15.8	14.3	1.3		68.6
CNF/SnO_2/PPy	59.9	9.1	14.3	16.7	
CNF/ Fe_2O_3/PPy	50.4	8.8	12.3		28.5

[a] Elemental compositions of samples was obtained using the EDS mode installed in the FE-SEM system (beam current: 10.0 μA, accelerating voltage: 10.0 kV).

Table S2. Comparison of the specific capacitances of CNF/SnO_2/PPy- and CNF/Fe_2O_3/PPy-based electrodes with previous studies.

Electrode Material	Potential Window	Current Density	Specific Capacitance	Reference
NCO-CNF	0 to 0.5 V	1.0 mA cm^{-2}	343.0 F g^{-1}	[1]
MnO-CNF	−0.2 to 0.9 V	0.5 A g^{-1}	246.0 F g^{-1}	[2]
N-HPCNF	0 to 0.6 V	1.0 A g^{-1}	394.0 F g^{-1}	[3]
3D-BN-CNF-ZF900	−1.0 to 0 V	0.5 A g^{-1}	295.0 F g^{-1}	[4]
MnO_2/CNF-CNT	0 to 1.0 V	0.5 A g^{-1}	483.5 F g^{-1}	[5]
CNF/SnO_2/PPy	−0.3 to 0.7 V	1.0 A g^{-1}	508.1 F g^{-1}	This work
CNF/Fe_2O_3/PPy	−1.0 to 0 V	1.0 A g^{-1}	426.8 F g^{-1}	This work

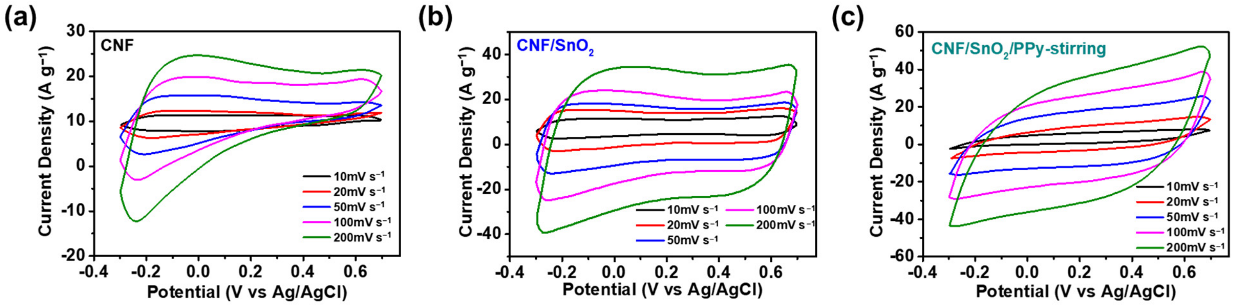

Figure S5. CV curves of (a) CNF-, (b) CNF/SnO_2-, and (c) CNF/SnO_2/PPy-stirring-based electrodes at various scan rates from 10 to 200 mV s^{-1} in the potential range of −0.3–0.7 V.

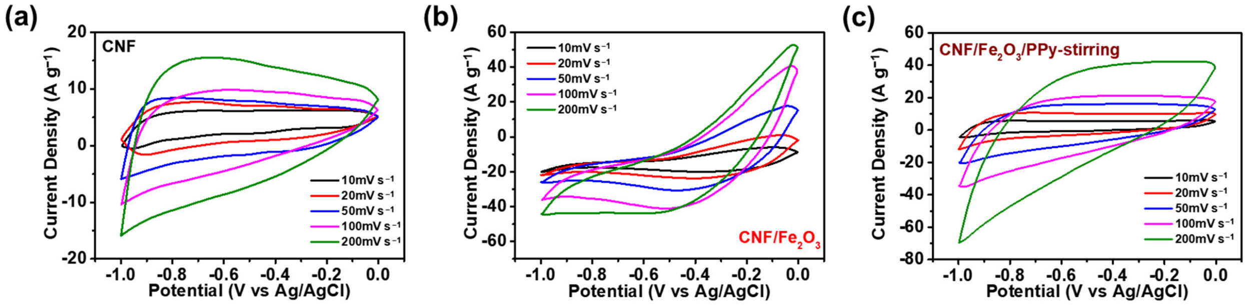

Figure S6. CV curves of (a) CNF-, (b) CNF/Fe_2O_3-, and (c) CNF/Fe_2O_3/PPy-stirring-based electrodes at various scan rates from 10 to 200 mV s^{-1} in the potential range of −1.0–0 V.

References

1. Xu, Z.; Gao, R.; Tao, Y.; Hou, X.; Cao, L. Three-dimensional $NiCo_2O_4$ nanosheets arrays on carbon nanofibers for high-performance asymmetric solid-state supercapacitor. *Diam. Relat. Mater.* **2021**, *119*, 108584.
2. Radhakanth, S.; Singhal, R. In-situ synthesis of MnO dispersed carbon nanofibers as binder-free electrodes for high-performance supercapacitors. *Chem. Eng. Sci.* **2023**, *265*, 118224.
3. Nie, H.; Mi, K.; Song, L.; Zheng, X. Nitrogen-doped hierarchical porous CNF derived from fibrous structured hollow ZIF-8 for a high-performance supercapacitor electrode. *RSC Adv.* **2019**, *9*, 40636–40641.
4. Dahal, B.; Mukhiya, T.; Ojha, G.P.; Muthurasu, A.; Chae, S.-H.; Kim, T.; Kang, D.; Kim, H.Y. In-built fabrication of MOF assimilated B/N co-doped 3D porous carbon nanofiber network as a binder-free electrode for supercapacitors. *Electrochim. Acta* **2019**, *301*, 209–219.
5. Huang, C.-L.; Chiang, L.-M.; Su, C.-A.; Li, Y.-Y. MnO_2/carbon nanotube-embedded carbon nanofibers as core-shell cables for high performing asymmetric flexible supercapacitors. *J. Ind. Eng. Chem.* **2021**, *103*, 142–153.
